# The Relationship Between Dog-Related Factors and Owners' Attitudes Toward Pets: An Exploratory Cross-Sectional Study in Korea

**DOI:** 10.3389/fvets.2020.00493

**Published:** 2020-08-18

**Authors:** Woo-Hyun Kim, Kyung-Duk Min, Sung-il Cho, Seongbeom Cho

**Affiliations:** ^1^College of Veterinary Medicine and Research Institute for Veterinary Science, Seoul National University, Seoul, South Korea; ^2^Department of Public Health Science, Graduate School of Public Health, Seoul National University, Seoul, South Korea; ^3^Institute of Health and Environment, Seoul National University, Seoul, South Korea

**Keywords:** dog ownership, exploratory study, human–animal bonds, Korean pet culture, Pet Attitude Scale

## Abstract

In Korea, there is a need for research on human–animal relationships because of an increase in the number of companion animals and the positive changes in public perception toward them. Few studies have examined these changes. This epidemiological study investigates the characteristics of Korean dog owners and their pet dogs and identifies the owner- and dog-dependent factors that influence the owners' attitudes toward pets. We conducted a cross-sectional study of dog owners by asking them to complete a Pet Attitude Scale-based questionnaire about their dogs and themselves. The participants included 654 young adults between 19 and 39 years of age who lived in Seoul and owned dogs. We found that most dogs were owned by single, educated, high-income men who preferred small purebred dogs. Most were also likely to underestimate their dog's body condition score (BCS). The multivariable logistic regression (odds ratio, OR) and the multiple linear regression (unstandardized coefficients, *B*) models suggested that positive pet attitudes were associated with nine factors: overweight (*OR* = 2.68, *B* = 5.28) or a normal BCS (*OR* = 2.09, *B* = 5.58), having a medical history of related diseases (*OR* = 2.36, *B* = 6.38) and vaccination (*OR* = 2.10, *B* = 6.22), buying the pet dog (*OR* = 0.60, *B* = −3.85), having a small dog (≤10 kg) (*OR* = 1.66), visiting the veterinarian frequently (*OR* = 1.08, *B* = 0.39), spending more time with the dog (*OR* = 1.23, *B* =1.32), and keeping other species in the house (*B* = −4.27). This study is the first to identify the relationships between owner- and dog-dependent factors and pet owner attitude toward pets, all within a Korean cultural context. This study highlights the factors associated with the development of relationships between pet dogs and their owners. The exploratory study is novel because it examines pet ownership in the context of the Korean culture; previous pet ownership studies were set in the West and are analyzed with Western cultural values in mind.

## Introduction

How relationships between humans and dogs are formed and maintained and what the results of these relationships are have attracted extensive scientific interest. This is because such relationships are closely related to the health and well-being of both animals and humans (1). Furthermore, it is generally believed that companion animals convey mental and physical health benefits to their human owners (2–4). Studies have found that owning and interacting with dogs benefits the dog owners' physical and psychological health (5–9). Meanwhile, dog owners provide their companions with food, shelter, companionship, and veterinary care, all of which benefit the dogs involved (10). Several studies have shown that dogs can benefit from physical interactions with their owners. For example, when companion animals are petted, their blood pressure and heart rates drop (11, 12). However, although most human and companion dog relationships are successful, some do fail, leading to the dog's abandonment and/or relinquishment (13). It is important to know which people keep dogs on a long-term basis, how they bond emotionally with their dogs, and what human- and dog-related factors may affect human–dog relationships.

Pet owners' demographic and socio-economic characteristics have been extensively investigated in Italy, Ireland, New Zealand, the United Kingdom (UK), the United States (USA), and Australia, with the objective of understanding human–animal bonds (14–22). Although the studies' variables and the associations evaluated differ greatly, factors such as age, gender, income/social class, marital status, rural/urban residence, and household type have been associated with pet ownership.

Furthermore, many researchers have attempted to develop standardized measures for assessing relationships between humans and their animals. The Pet Attitude Scale (PAS) was developed as a quantitative scale for assessing human attitudes toward their pets (23). PAS has also been used to develop other human–animal bond measures such as the Companion Animal Bonding Scale (24) and the Lexington Attachment to Pets Scale (25). Previous research on dog ownership using PAS focused on different aspects of human–animal bonds such as the genesis of pet attitudes in families (26), reduced loneliness (27), and improvements to immune system functions (28).

Dogs have become an integral part of the Korean society, and the number of pet owners is growing. It has been estimated that 30.9% of Korean households, 5.9 million households, currently have a dog as a companion animal (29). In Korea, dog consumption continues, but over the past 15 years there has been a cultural shift in public attitudes toward dogs as pets (30). Dogs as companions did not become commonplace in Korea until after the 1990s when the economic situation improved. Korea's pet ownership culture has been in vogue for a shorter period than in the Western culture (31). In particular, this consciousness change about companion animals is occurring in the younger generation, aged 19–39 years. According to a survey, 32.3% of the younger generation said that they currently keep companion animals (32). Furthermore, most Korean people surveyed (63.5%) agree that a companion animal is a family member (29). In light of this phenomenon, there is a growing need for research on the relationships between dogs and people in Korea, especially with the younger generation.

Despite the importance of research on human–animal relationships in Korea, we were unable to locate published studies on Korean pet ownership demographics that focus on this relationship. Previous studies of human attitudes toward pets focused mostly on aspects of pet ownership such as education level, family size, and gender (33, 34). Unlike these previous studies that examined existing socio-economic factors, this study investigates pet owner behaviors, including dog acquisition and subsequent veterinary care. In this exploratory study, we assume that human attitudes toward pets are associated with factors related to both the dogs and their owners. The aim of this cross-sectional study is to first document the characteristics of Korean dog owners and their dogs and then use the PAS to analyze these data and identify the owner- and dog-dependent factors that influence the owners' attitudes toward pets. This exploratory study will also pave the way for the study of the relationship between humans and companion animals in Korea.

## Methods

### Subject

The survey used to conduct this research was designed and constructed by Research and Research, Inc., in Seoul, South Korea. The R panel (http://panel.randr.co.kr/) consists of a pre-built panel group developed by Research and Research to conduct an email survey of the Korean population for statistical research, consisting of approximately 1.13 million South Koreans over 14 years old. In this panel, we selected young adults between 19 and 39 years of age who were living in Seoul and randomly emailed 25,000 registered panel members. We sent questionnaires via email to the selected pet owners from September to October 2017. We received a total of 1,040 survey responses from individuals who were willing to participate in this study. We included only pet owners currently living with dogs; dog owners who were raising dogs for food or for breeding purposes were excluded. A total of 654 dog owners participated in this study.

### Data Collection and Questionnaire

The questionnaire, which gathered information about regular health care for dogs, was developed by the Australian Veterinary Association (35) and was informed by two clinical veterinarians. It included both open and closed questions pertaining to the owners' and the pets' demographics. The online survey consisted of four sections and included 46 binary, multiple choice, and short answer type questions (see Appendix A in Supplementary Material).

The first section asked questions about the pet owners' general characteristics, such as gender, age, marital status, children, general health, educational level, employment, annual income, and the type of house that the owner is living in.

The second section pertained to pet ownership, such as the age of first pet ownership, the number of dogs, age of the oldest dog, whether there were other species of pets in the house, amount of time the dog has been owned, and the source of acquisition.

The third section gathered information on the demographics and the health of the pets, such as gender, whether they were altered, size, breed, nine-point body condition score (BCS), frequency of exercise per day, length of time that the owners spent with their dogs per day, number of veterinary visits in a year, and the dog's disease history for 1 year. If a dog visited the hospital several times with the same disease, it was recorded as one in the dog's disease history.

The fourth section comprised a standardized, back-translated Korean language version of the Modified Pet Attitude Score (PAS) (36). The PAS uses 18 questions based on a seven-point Likert scale to measure the respondent's attitudes toward pets: 1 = strongly disagree, 2 = moderately disagree, 3 = disagree, 4 = unsure, 5 = slightly agree, 6 = moderately agree, and 7 = strongly agree.

### Statistical Analyses

We investigated the distribution according to the socio-economic factors of our pet owner sample population and compared it with the younger generation (19–39 years) of Seoul to identify the characteristics of the participants. In this analysis, we used the Mantel–Haenszel chi-square, wherein a score of 0.05 was used as the cutoff for significance (37). The demographic data of Seoul included gender, age, marital status, education level, employment, annual income, and type of dwelling. These data were obtained from the Korean Statistical Information Service and were compared with the demographic data from the sample population (http://kosis.kr).

For the variable “age of first pet ownership,” the three categories for those under 19 years of age were merged into one category, and the variables were separated into “juvenile” and “adult” groups. The variable “number of dogs in the house” was changed to “there is another dog in the house.” The variable “source” was changed to the dichotomous variable “buying the dog.” The categories “pet shop,” “breeder,” “veterinarian,” and “Internet” were merged under “buying the dog: Yes.” The miniature and the small categories were merged under “small,” and more than 10 kg was integrated into “large.” The nine-scale BCSs were integrated into underweight (1–3), normal (4–5), and overweight (6–9). To estimate the PAS, questions were classified as both positive and negative. Six negative questions were reverse-coded: “Having pets is a waste of money,” “I feel that pets should always be kept outside,” “The world would be a better place if people would stop spending so much time caring for their pets and started caring more for other human beings instead,” “Animals belong in the wild or in zoos, but not in the home,” “Pets are fun, but it's not worth the trouble of owning one,” and “I hate animals.”

The dependent variable was analyzed both as a binary variable, using logistic regression (model 1), and as a continuous variable, using multiple linear regression (model 2). In both analyses, there was a univariate analysis and a subsequent multivariable analysis. Factors with a *p* ≤ 0.20 in the univariate analysis were considered for inclusion in a multivariable analysis, and the variables with a significance level of *p* ≤ 0.05 were retained in the final model. For logistic regression, the participants were separated into high- and low-PAS groups based on the mean value of 90 points. Logistic regression was used to conduct an initial univariate analysis. Correlation analysis was used to test for collinearity among the variables to be included in the multivariable analysis. Multivariable logistic regression with a forward stepwise variable selection was used. The model's fit was evaluated using the Hosmer–Lemeshow test (38). The proportion of variance in dependent variables associated with the variables was explained by the Nagelkerke *R*^2^ value (39). For multiple linear regression, the univariate analysis was carried out using Pearson correlation and ANOVA. Multiple linear regression analysis was carried out using stepwise variable selection. Autocorrelation was tested using the Durbin–Watson test (40). Adjusted *R*^2^ was used to explain the proportion of the variance explained by the variables. The effects of the independent variables are shown as odds ratio (OR) for the logistic regression analysis and as unstandardized coefficients (*B*) for multiple linear regression.

All statistical analyses were performed using SPSS v. 23.0 (IBM, Armonk, NY, USA) and R software version 3.4.3 (R Project for Statistical Computing, Vienna, Austria).

## Results

### Demographics of Dog Owners

The results of the demographics of dog owners compared to the general population of Seoul are presented in Table 1. The demographics were different for all the variables studied between dog owners and the general population.

**Table 1:** Demographics of dog owners in Seoul, South Korea.

**Variables**	**Category**	**Pet owner**		**Seoul population^a^**		***P*-value**
		***N***	**%**	***n***	**%**	
Total		654	100.00%	2,940,284	100.00%	
Gender						0.005
	Male	362	55.35%	1,467,482	49.91%	
	Female	292	44.65%	1,472,802	50.09%	
Age	Mean: 32.14 (*SD*: 4.89)					<0.001
	19–29 years old	178	27.22%	1,394,291	47.42%	
	30–39 years old	476	72.78%	1,545,993	52.58%	
Marital status						<0.001
	Yes	333	50.92%	1,901,290	64.66%	
	No	321	49.08%	1,038,994	35.34%	
Children						<0.001
	Yes	271	41.44%	1,793,790	61.01%	
	No	383	58.56%	1,146,494	38.99%	
Education	University graduate					<0.001
	Yes	619	94.65%	2,487,949	84.62%	
	No	35	5.35%	452,336	15.38%	
Employment						<0.001
	Employed	509	77.83%	1,652,440	56.20%	
	Self-employed	46	7.03%	378,415	12.87%	
	Help family affair	13	1.99%	52,337	1.78%	
	Unemployed	86	13.15%	857,093	29.15%	
Annual income						
	Quartile					<0.001
	Q1 (lowest) (≤1,800)	116	17.74%	596,878	20.30%	
	Q2 (>1,800 ≤3,000)	141	21.56%	1,114,368	37.90%	
	Q3 (>3,000 ≤4,200)	194	29.66%	611,579	20.80%	
	Q4 (highest) (>4,200)	203	31.04%	617,460	21.00%	
House type						<0.001
	Detached house	61	9.33%	358,715	12.20%	
	Apartment	399	61.01%	1,704,777	57.98%	
	Multi-family house	179	27.37%	726,544	24.71%	
	Studio apartment	15	2.29%	150,249	5.11%	

a *Population data taken from the Korean Statistical Information Service (http://kosis.kr/index/index.do)*.

### Factors Related to Dog Ownership Pattern in the Study's Dog Owners

Table 2 reveals the categorical variables related to pet ownership which were obtained in the questionnaire. There was an almost equal split between juvenile dog acquisition (50.31%) and adult dog acquisition (49.69%). In 178 responses (27.22%), there were also other species in the house, and 525 pet owners reported having only one dog in the house (80.28%). Because of a low response rate (19.78%), the variables related to the dogs' ages (mean: 6.29 years, *SD*: 5.81 years) and the amount of time owned (mean: 5.47 years, *SD*: 3.82 years) were not included in the univariable and multivariable models. A total of 286 pet owners (43.73%) reported that they bought the dog.

**Table 2:** Results of the categorical variables related to pet ownership which were obtained through the questionnaire distributed to Seoul citizens between the ages of 19 and 39 (*n* = 654).

**Variables**	**Category**	**Total number**	**Percentage**
**Age of first pet ownership**			
	<6 years old	64	9.79%
	6–12 years old	134	20.49%
	13–18 years old	131	20.03%
	Above 19 years old	325	49.69%
**Other species in the house**			
	Yes	178	27.22%
	No	476	72.78%
**Numbers of dogs in the house**			
	1	525	80.28%
	2	103	15.75%
	3	18	2.75%
	≥4	8	1.22%
**Source**			
	Adopted from shelter	37	5.66%
	Strayed in	22	3.36%
	Pet shop	228	34.86%
	Breeder	52	7.95%
	Friend or gifted	308	47.09%
	Born in house	1	0.15%
	Veterinarian	3	0.46%
	Internet	3	0.46%

### Demographics of Dogs and Their Health-Related Factors

Table 3 summarizes the categorical variables of the dogs and their health-related factors. Most dogs were purebred (76.76%) small dogs (82.75%) that weighed between 2 and 10 kg. Approximately 65.75% of the dog owners estimated that owner-reported perception of BCS was in the normal range (4–5). In addition, 20.49% of the participants perceived their dog as underweight and 13.75% recognized it as overweight. When the dog owners were asked how often they exercised their dogs, 20.80% said “once a day,” 48.01% said “once every 2 or 3 days,” and 16.36% said “once every 4–6 days.” These results indicate that most owners usually exercise their dogs at least once a week (85.02%). The length of time spent exercising their dogs was 63.92 ± 44.13 min per day (mean ± SD), and the time spent with their dogs was 4.19 ± 3.98 h per day. The dog owners visited the veterinary hospital 4.47 ± 5.42 times per year, and 43.12% of the dog owners visited a hospital more than four times a year. The dogs' medical history for a 1-year period included the following: vaccinations (14.96%), skin conditions (13.84%), dental work (13.20%), intestinal issues (8.64%), ophthalmological issues (7.52%), treatment for heartworm (7.20%), respiratory issues (6.48%), orthopedic conditions (4.64%), cardiac symptoms (2.80%), emergencies (2.56%), cancer (1.52%), neurological symptoms (1.28%), and other (0.64%).

**Table 3:** Results of the categorical variables related to the characteristics of pet dogs which were obtained through the questionnaire distributed to Seoul citizens between the ages of 19 and 39 years old (*n* = 654).

**Variable**	**Total number**	**Percentage**
**Breed**		
Pure breed	502	76.76%
Cross breed	152	23.24%
**Sex and neutralization**		
Male: castration	246	37.61%
Male: no castration	134	20.49%
Female: spray	118	18.04%
Female: no spray	156	23.85%
**Size**		
Mini (<2 kg)	11	1.68%
Small (2–10 kg)	540	82.57%
Medium (11–25 kg)	90	13.76%
Large (25–50 kg)	11	1.68%
Giant (>50 kg)	2	0.31%
**Frequency of exercising with the dog**		
Once a day	136	20.80%
Once every 2 or 3 days	314	48.01%
Once every 4–6 days	107	16.36%
Once a week	79	12.08%
No walking	18	2.75%
**Dogs' medical history for a 1-year period**		
None	184	14.72%
Vaccination	187	14.96%
Skin	173	13.84%
Dental	165	13.20%
Intestine	108	8.64%
Ophthalmology	94	7.52%
Heartworm	90	7.20%
Respiratory	81	6.48%
Orthopedic	58	4.64%
Cardiac	35	2.80%
Emergency	32	2.56%
Cancer	19	1.52%
Neurologic	16	1.28%
Other	8	0.64%

### Pet Owners' Pet Attitude Scale

The PAS mean value was 90 points, with a standard deviation of 12.87 (Table 4). The three items with the highest mean rankings were “I love pets,” “I really like seeing pets enjoy their food,” and “House pets add happiness to my life (or would if I had one).” The higher PAS scores indicate that pet owners have more favorable and supportive attitudes toward their pets.

**Table 4:** Owners' attitudes toward their pets based on the Pet Attitude Scale (PAS).

**PAS item**	**Positive/negative^a^**	**Mean**	***SD***
I really like seeing pets enjoy their food	Positive	5.60	1.35
My pet means more to me than any of my friends (or would if I had one)	Positive	4.66	1.31
I would like to have a pet in my home	Positive	5.29	1.18
Having pets is a waste of money	Negative	5.13	1.47
House pets add happiness to my life (or would if I had one)	Positive	5.47	1.22
I feel that pets should always be kept outside	Negative	4.54	1.46
I spend time every day playing with my pet (or would if I had one)	Positive	5.07	1.18
I have occasionally communicated with my pet and understood what it was trying to express (or would if I had one)	Positive	5.10	1.11
The world would be a better place if people would stop spending so much time caring for their pets and started caring more for other human beings instead	Negative	3.65	1.36
I like to feed animals out of my hand	Positive	5.25	1.17
I love pets	Positive	5.74	1.15
Animals belong in the wild or in zoos, but not in the home	Negative	4.71	1.59
If you keep pets in the house, you can expect a lot of damage to furniture	Positive	4.00	1.53
I like house pets	Positive	5.29	1.16
Pets are fun but it's not worth the trouble of owning one	Negative	4.20	1.49
I frequently talk to my pets (or would if I had one)	Positive	5.36	1.19
I hate animals	Negative	5.51	1.49
You should treat your house pets with as much respect as you would a human member of your family	Positive	5.42	1.13
Total score of PAS		90.00	13.90

*The PAS was assessed using the following Likert scale: 1, strongly disagree; 2, moderately disagree; 3, disagree; 4, unsure; 5, slightly agree; 6, moderately agree; 7, strongly agree; SD, standard deviation*.

a *Direction of coding scale: negative means that the score was reverse-coded*.

### Univariable Analysis of Variables

As shown in Table 5, using univariable analysis, eight variables were identified as the factors associated with high PAS in model 1 and model 2. In categorical data, the pet owners who perceived and reported their dog as overweight (*OR* = 2.91, *B* = 8.16) or to have a normal weight (*OR* = 2.27, *B* = 6.91) had more positive attitudes toward their pets than the owners who perceive them as underweight. Regarding a pet's medical history over 1 year, pet owners whose pets had visited the hospital because of a disease (*OR* = 2.47, *B* = 6.62) or to receive a vaccination (*OR* = 2.35 *B* = 7.33) had a higher PAS than owners who did not have any record of a disease history in a 1-year period. Pet owners with high PAS were associated with three factors: “small dogs” (≤10 kg; *OR* = 1.70, *B* = 3.13), “not keeping other species in the house” (*OR* = 1.71, *B* = 4.75), and “not buying a dog” (*OR* = 1.39, *B* = 2.78). In continuous variables, the participants who spent more time with the dog (*OR* = 1.24, *B* = 1.43) and visited a veterinarian frequently (*OR* = 1.13, *B* = 0.72) showed relatively high PAS scores. However, the participants who spent a longer time with the dog for exercise showed relatively low PAS scores (*OR* = 0.99, *B* = −0.05).

**Table 5:** Results of the univariable logistic regression (model 1) and linear regression (model 2) of the variables associated with the high Pet Attitude Scale (PAS).

**Variables**	**High PAS^a^*n* = 339 (%)**	**Low PAS^a^*n* = 315 (%)**	**Total *n* = 654**	**Model 1**			**Model 2**		
				**OR**	**95% CI**	***P*-value**	***B***	**95% CI**	***P*-value**
**BCS**									
Overweight (6–9)	55 (61.11)	35 (38.89)	90	2.91	1.67–5.06	<0.001	8.16	4.50–11.82	<0.001
Normal (4–5)	237 (55.12)	193 (44.88)	430	2.27	1.52–3.40	<0.001	6.91	4.26–9.59	<0.001
Underweight (1–3)	47 (35.07)	87 (64.93)	134	–		<0.001	–		
**Pet's medical history over a year**									
Disease	235 (58.31)	168 (41.69)	403	2.47	1.73–3.53	<0.001	6.62	4.26–8.99	<0.001
Vaccination	36 (57.14)	27 (42.86)	63	2.35	1.32–4.21	<0.001	7.33	3.43–11.23	<0.001
None	68 (36.17)	120 (63.83)	188	–		<0.001	–		
Spending more time with dog	Mean: 4.23 h	*SD*: 3.98 h	654	1.24	1.17–1.31	<0.001	1.43	1.18–1.68	<0.001
Frequently visiting a veterinarian	Mean: 4.47 times	*SD*: 5.42 times	654	1.13	1.08–1.18	<0.001	0.72	0.53–0.91	<0.001
Exercise time with dog	Mean: 63.8 min	*SD*: 44.13 min	654	0.99	0.98–0.99	0.001	−0.05	−0.08 to −0.02	0.001
**Size**									
Small (≤10 kg)	297 (53.90)	254 (46.10)	551	1.70	1.11–2.60	0.002	3.13	0.19–6.06	0.037
Large (>10 kg)	42 (40.78)	61 (59.22)	103	–			–		
**Keeping other species in the house**									
No	264 (55.46)	212 (44.54)	476	1.71	1.21–2.42	0.003	4.75	2.37–7.13	<0.001
Yes	75 (42.13)	103 (57.87)	178	–			–		
**Buying the dog**									
No	204 (55.43)	164 (44.57)	368	1.39	1.02–1.97	0.037	2.78	0.62–4.93	0.012
Yes	135 (47.20)	151 (52.80)	286	–			–		

a *The mean value of 90 was used as the criterion for classifying PAS scores into high or low groups*.

*Model 1 is the result of a univariable logistic regression in the high-PAS group where the dependent variable was considered as a categorized variable based on the mean value of 90. Model 2 is the result of a simple linear regression in the high-PAS group where the dependent variable was considered as a continuous variable*.

*OR, odds ratios; B, unstandardized beta; CI, confidence interval*.

### Multivariable Analysis of Variables

Eight variables with *p* ≤ 0.2 were used to estimate the effects using the multivariable logistic regression model and the multiple linear regression model (Table 5). The final logistic regression model, model 1, identified six variables as independent variables for pet owners with high PAS (Table 6). In category variables, they were “BCS: overweight” (*OR* = 2.51, 95% CI 1.38–4.54) and “normal” (*OR* = 2.16, 95% CI 1.41–3.32) and “pet's medical history: disease” (*OR* = 2.36, 95% CI 1.57–3.55), and “vaccination” (*OR* = 2.10, 95% CI 1.12–3.94). In dichotomous variables, they were “buying the dog” (*OR* = 0.60, 95% CI 0.42–0.85) and “small dog” (*OR* = 1.78, 95% CI 1.09–2.90). Finally, in continuous variables, they were “frequently visiting the veterinarian” (*OR* = 1.08, 95% CI 1.03–1.13) and “spending more time with the dog” (*OR* = 1.23, 95% CI 1.16–1.32). Model 1 was statistically significant [$\chi_{\left(9\right)}^{2}$ = 153.176, *p* < 0.001], and the model explained 27.9% (Nagelkerke *R*^2^) of the variance in the high-PAS group.

**Table 6:** Results of the multivariable logistic model and linear regression of the variables associated with the high Pet Attitude Scale (PAS).

**Variables**	**Model 1**			**Model 2**		
	**OR**	**95% CI**	***P*-value**	***B***	**95% CI**	***P*-value**
**BCS**						
Overweight	2.68	1.43–5.01	0.002	5.28	2.00–8.53	0.002
Normal	2.09	1.34–3.26	0.001	5.58	3.22–7.94	<0.001
Underweight	–			–		
**Pet's medical history over a year**						
Disease	2.36	1.57–3.55	<0.001	6.38	4.25–8.50	<0.001
Vaccination	2.10	1.12–3.94	0.021	6.22	2.75–9.29	<0.001
None	–			–		
**Buying the dog**						
Yes	0.60	0.42–0.85	0.005	−3.85	−4.74 to −0.97	0.003
No	–			–		
**Size**						
Small (≤10 kg)	1.78	1.09–2.90	0.022	–		
Large (>10 kg)	–			–		
Frequently visiting the veterinarian	1.08	1.03–1.13	0.002	0.39	0.21–0.57	<0.001
Spending more time with the dog	1.23	1.16–1.32	<0.001	1.32	1.09–1.56	<0.001
Keeping other species in the house	–			−4.27	−6.38 to −2.16	<0.001
*R*^2^	0.279			0.274		

*Model 1 is the result of a multivariable logistic regression in the high-PAS group where the dependent variable was considered as a categorized variable based on the mean value of 90. Model 2 is the result of a multiple linear regression in the high-PAS group where the dependent variable was considered as a continuous variable. In model 1, the Nagelkerke R^2^ value was estimated, and in model 2, the adjusted R^2^ value was estimated*.

*OR, odds ratios; B, unstandardized beta; CI, confidence interval*.

The results of the multiple linear regression in model 2 are shown in Table 6. The model was significant with *F*_(7, 646)_ = 36.245, *p* < 0.001, and adjusted *R*^2^ = 27.4%. The variables included in the model were “BCS: overweight” (*B* = 5.28, 95% CI 2.00–8.53) and “normal” (*B* = 2.16, 95% CI 1.41–3.32), “pet's medical history: disease” (*B* = 6.38, 95% CI 4.25–8.50) and “vaccination” (*B* = 6.22, 95% CI 2.75–9.29), “buying the dog” (*B* = −3.85, 95% CI −4.74 to −0.97), “frequently visiting the veterinarian” (*B* = 0.39, 95% CI 0.21–0.57), “spending more time with the dog” (*B* = 1.32, 95% CI 1.09–1.56), and “keeping other species in the house” (*B* = −4.27, 95% CI −6.38 to −2.16).

## Discussion

This research aims to investigate the characteristics of Korean dog owners and their dogs. Furthermore, the study identified how our participants' attitudes toward pets are associated with owner- and dog-dependent variables. This is the first explanatory cross-sectional study to document Korean pet dogs and their owners' characteristics and demographics. The results highlight the factors associated with the development of a relationship between a dog and its owner. It is also expected that a comparison between East-Asian and Western pet cultures will advance the socio-cultural pet research.

### Study Participants' Demographics

Comparing our study sample's population with the general population revealed some characteristics of our pet owners' population. As shown in Table 1, the study participant population tended to be male, older, university-level educated, employed, and earning high incomes within the Seoul population. This tendency might be due to the fact that the study participants were limited to individuals who own dogs and could easily access the Internet to complete the questionnaires. These differences are likely to be underestimated as the general population also includes pet owners.

This study found that males were more likely to participate in the survey and be interested in pet ownership (55.35% of respondents) than females and that the proportions of male and female owners were similar to those reported in previous pet ownership studies (17, 23, 41, 42). A cross-sectional study of dog ownership in Tanzania also found that male-headed households were more likely to have a dog (17). In a telephone survey in Taiwan, Hsu et al. found that male respondents were more likely than females to report ever having owned a dog (41). On the PAS and CABS, males had a more positive attitude toward pets than did females (23, 42). Several North American and European studies, however, found that females generally have a higher participation rate and degree of attachment to their companion animals (14, 15, 19, 43, 44). In another study, gender differences in relation to peoples' attachment to pets were generalized, depending on age, species, and national factors (45).

Compared with national demographic data, the sample population had a statistically higher proportion of couples without children or unmarried individuals. Some pet ownership studies in Western countries such as Ireland (15), the UK (19), New Zealand (46), and the USA (47) have suggested that pets are more common in families with children than in families without children. However, our study, which comprised young adults, was different from the Western pet studies which used a broader population range. Moreover, Seoul has a lower proportion of families with children in our sample population than in the Western studies. Other studies report similar findings: single people or couples without children represent a high proportion of pet owners (33) and have strong emotional bonds with their dogs (48).

There were statistical differences between the socio-economic factors of the study population and the general Korean population, including education level, employment, and income. Most study participants were university graduates (94.65%); this rate is higher than that for the general Korean population and of other countries like Australia (62%) (49) and Spain (57.71%) (33). Moreover, income levels and the proportion employed were also higher than those of the total population. These socio-economic factors—education level, employment, and income—seem to be intertwined. A study of Spanish pet owners found that highly educated people were more likely to have a pet, and pet owners' scores on Cantril's Self-Anchoring Ladder were higher than those reported for the Spanish average (33). Some studies of pet owners in the USA, Brazil, and the Netherlands found that higher-income households were more likely to have a dog than lower-income households (50–52). However, other studies reported that households with higher education level were less likely to own a dog than those with lower levels (19). There is also the possibility that our sampling was biased. The more educated a person was, the more interested they were in the survey or the more time they spent answering it sincerely.

### Characteristics of Dogs and Pet Owners With High PAS

We used multivariable logistic regression and multiple linear regression to identify the factors contributing to the study participants' high PAS scores and found associations between owners' PAS and several owner- and dog-related characteristics (Table 6).

In the final results of our multivariable analysis, dog size was associated with a high PAS (*OR* = 1.78, 95% CI: 1.09–2.90). These results are consistent with a previous study which found that the owners' perceptions of dog behavior were related to dog size and to the extent the owners shared activities with their dogs; it also noted that owner interaction increased the smaller the dog was (53). This may be explained by the “canine cuteness effect,” where pet owners have a stronger relationship with dogs perceived as cute (54).

In our study, 84.25% of pet owners had small dogs (2–10 kg) and/or miniature dogs (<2 kg) (Table 3). Compared with the preferred dog size in other countries such as Spain (39.0%) (33), Australia (50.3%) (20), and Italy (39.4%) (14), the Korean pet owners in our study preferred small pet dogs. Korea's unique residential environment might contribute to the large proportion of small dogs. According to Korea's Ministry of Land, Infrastructure, and Transportation, the housing space per person is 31.2 m^2^, which is less than in the USA, the UK, and Japan (55). Most pet owner respondents also lived in apartments (61.0%), where limited space would make it difficult to satisfy a large dog's physical requirements.

Owner attitudes were associated with the animal's size but not with breed type. There was no association between owner's PAS and the variable “purebred.” Previous studies also reported no differences in owners' attitudes and commitment (time playing with their dog and reason for pet ownership) between crossbred dogs and purebred dogs (56). Our sample reported 76.76% for purebred, which is high compared to those of other countries, 47% in the USA and 67–69% in the UK (57, 58). The owners of small and purebred dogs represent a high proportion of our sample population.

The results of our multivariable analysis confirmed that the owners who perceived their own dog as overweight (*OR* = 2.68, *B* = 5.28) or of normal weight (*OR* = 2.09, *B* = 5.58) scored higher on the PAS than the owners of who perceived their dog as underweight. These results imply that the respondents have a more positive attitude when they perceive their dog as overweight or normal, or it could also suggest that pet attitudes could be related to the dog weight perception. This might be associated with whether their positive pet attitude had been formed through the feeding process or *vice versa*. Additionally, the owners with a positive pet attitude may feed their dogs more frequently, hence the higher BCS. In our survey, two statements related to feeding—“I really like seeing pets enjoy their food” and “I like to feed animals out of my hand”—showed higher than mean scores (Table 4). Bland et al. previously suggested that households with overweight dogs fed their dogs more treats and offered food in different patterns than did owners of normal-weight dogs (59).

Our study's distribution of dog BCS was quite different from the distribution reported in other countries' studies. Our respondents indicated that 65.75% of their dogs had a normal BCS, while 20.49% considered their dogs as underweight, and only 13.76% thought that their dogs were overweight. A survey of pet owners' feeding practices in the USA and Australia reported a similar distribution of normal BCS but found that about 30.6% of the dogs were overweight and only 4.1% were underweight (60). We hypothesize that the differences between the sizes of dogs in Korea and other countries might explain the large distribution of underweight dogs in our study population. The national scale-based study conducted in the USA has reported that large breeds are at a greater risk of obesity (61). Another hypothesis of the BCS distribution would be the owners' misperceptions of their dogs' BCS. The owners in this study were given a copy of the nine-point BCS chart and asked to assess their dogs' BCS. Recent studies show that most owners misperceive their dog's body condition. In particular, 44.1% of pet owners underestimated their dog's BCS (62–64), and the owners' misperceptions persisted even when they referred to a BCS chart (63). A cross-sectional study in Glasgow found that the prevalence of misperception was likely to be closely related to the prevalence of obesity in a population (64).

In the final multivariable model, pet owners who visited the veterinary hospital frequently had a more positive attitude toward pets than owners whose veterinary visits were less frequent (*OR* = 1.08, *B* = 0.39). This suggests a complementary relationship between an owner's attitude toward their pet and hospital visits. It is possible to assume that owners who have a friendly attitude toward their pets are more likely to be concerned about their pet's behavior and clinical symptoms and hence will visit the hospital more frequently. Other variables—“disease history: disease” (*OR* = 2.36, *B* = 6.38) and “disease history: vaccination” (*OR* = 2.10, *B* = 6.22)—were also selected in the final model. These results strongly support the hypothesis that there is a relationship between owner PAS and dog health-related factors.

Pet owners who spent more time with their dog had higher PAS scores than pet owners who spent few hours with their dog (*OR* = 1.23, *B* = 1.32). The results showed an association between the time spent with the dog and a positive attitude. This is probably related to the question “I spend time every day playing with my pet” in PAS (mean score: 5.47). Quality time spent with the dog has been associated with the seven dimensions of dog companionship in a study of 749 dog owners (65). However, some studies reported that spending a lot of active time with the dog could result in behavioral problems (20, 53).

Pet owners with other species of pets such as cats or exotic animals were less familiar with dogs than pet owners without other species of pets (*B* = −4.27). In the survey estimating the willingness of dog owners to keep other species, cats were the most common choice (27.9%) (32). Dogs and cats frequently have conflicts when they live in the same household due to their different communication signals and behaviors, which are misinterpreted by the other species (66). This situation could be stressful for pet owners and makes it difficult for dogs and their owners to develop a close relationship.

Pet owners who bought their dogs had a lower PAS than owners who had received their dog without buying it (e.g., it was adopted, a stray, from a friend, or gifted) (*OR* = 0.60, *B* = −3.85). In studies on risk factors associated with relinquishment, low incomes and cost were reported as factors related to relinquishment (67, 68). Relinquishment and pet attitude are similar in terms of forming and maintaining a relationship with the dog. This suggests that economic problems are an important factor in the relationship between dogs and their owners and may impact the level of bonding between the dog and the owner. In this study, almost half of the owners had been given dogs as gifts (47.1%), and about a third purchased their dogs at a pet shop (34.86%) or from a breeder (7.95%), adopted it from a shelter (5.66%), or found it as a stray (3.36%) (Table 2). Previous studies reported that, in Canada, 44% of dogs were acquired from a friend or family member (52), in Italy 48% (14), and in the USA 25% (69). A recent USA study found that 25% of dogs were given to their owners by friends, 25% were purchased from a breeder, 22% were adopted from a shelter or humane society, and 12% were adopted from rescue groups (69). As a result, the rate of purchase in pet shops is higher in Korea than in the West, and relatively few dogs are adopted from shelters.

### Study Limitations

There are several limitations in this study. First, this study is limited to young-generation adults living in urban areas in Seoul. The sample selection was based on the peculiarities of the Korean dog culture compared with the dog culture of the West. Other studies of pet ownership have included an older population in the UK and the USA. These countries have a long history of keeping dogs as pets, and the participants' age groups in these studies were higher than that in our study. In Korea, keeping a dog as a companion animal began in the 1990s, after the economy and the living standards improved (31). Until then, dogs were regarded more often as food. While the younger generation may reject the food culture, older generations still regard dogs as food (70). This tendency is especially acute in rural areas, and cases of eating lost dogs in the countryside are frequent in Korea (71). Therefore, young people in urban areas were the main targets of our investigation into the characteristics of human–animal bond culture and PAS.

Second, our self-report measures could lead to information bias and misclassification bias. For example, pet owner perception in BCS could exaggerate due to misperception, as mentioned above. Although we tried to secure objectivity by presenting the picture guide (S1), it is difficult for the owners to estimate the exact BCS by looking at the guide (63). Further study would be needed to evaluate the Korean dogs' BCS through experts who can determine BCS objectively.

Finally, the study results could not determine the causality and the direction between the PAS and identified variables assumed to be associated due to its cross-sectional design. The PAS and the variables can be associated in both directions, and either direction would have implications (i.e., pet owner's hospital visits can lead to positive pet attitudes or *vice versa*). Further studies are needed to investigate the causality and the direction, and it would bring a deeper understanding of the relationship between people and dogs.

### Conclusion

We analyzed and identified pet dogs' health- and ownership-related factors associated with Korean owners' attitudes toward pets. We found that pet owners were more likely to have a high PAS if the dog was perceived as overweight or of normal weight, the owner visited the veterinarian in case of disease or vaccination, had visited a veterinary hospital frequently, the owner spent more time with the dog, the dog was given as a gift rather than purchased, other species are kept in the house, or the dog weighed <10 kg. This study highlights the importance of a pet dog's health, size, and origin. Considering these factors could foster a more desirable relationship between humans and animals. Our results suggested that Korean pet owners prefer small, purebred dogs. We also reported the pet dogs' demographics and their owners' socio-economic status. These results may have implications for different types of pet ownership in different cultures and imply the need for further studies on pet ownership in other cultures. The findings from this study could be used to advance the cross-cultural validation of the PAS and inform future pet ownership studies in Korea.

## Data Availability Statement

All datasets generated for this study are included in the article/Supplementary Material.

## Ethics Statement

The Institutional Review Board (IRB) at the Seoul National University gave permission to conduct this study (IRB approval number: SNU IRB No. E1708/003-001). Survey participants were fully informed about the purpose and background of the study by e-mail. All survey data gathered were protected and processed anonymously.

## Author Contributions

W-HK designed the study, investigated the data collection, performed the data analysis, and participated in the manuscript preparation. K-DM designed the study, investigated the data collection, reviewed the data, and participated in the manuscript review. SuC designed the study, acquired funds, and participated in the manuscript review. SeC supervised the project, administrated the project, and participated in the manuscript review. All authors contributed to the article and approved the submitted version.

## Conflict of Interest

The authors declare that the research was conducted in the absence of any commercial or financial relationships that could be construed as a potential conflict of interest.
