# Supplementary material for: The Relationship Between Dog-Related Factors and Owners' Attitudes Toward Pets: An Exploratory Cross-Sectional Study in Korea
Source: Front Vet Sci. 2020 Aug 18;7:493. doi: 10.3389/fvets.2020.00493 (PMC7461997; doi:10.3389/fvets.2020.00493)
Supplement: Supplementary file 1 [file Data_Sheet_1.docx]

**Appendix A. Questionnaire for study participants: Dog owners**

**Overview**

**Introduction**

**Purpose of this questionnaire:** To identify characteristics of dog owners and their dogs

**Study participants:** Between 19 and 39 years of age, resident of Seoul (**3 Questions)**

| Category | Target | Contents | Number of questions |
| --- | --- | --- | --- |
| General characteristics | Pet owners | Gender, Age, Marital status, Children, General health, Education level, Employment status, Income, House type | 10 questions |
| General characteristics and health | Companion animals |  | 18 questions |
| Pet Attitude Scale (PAS) | Pet owner and companion animals | Pet attitude scale | 18 questions |

| **Study Participant** |
| --- |

**1. Is your age between 19 and 39?**

① Yes (→ Go to question 2)

② No (→ Unable to participate in the survey)

**2. Do you live in Seoul?**

① Yes (→ Go to question 3)

② No (→ Unable to participate in the survey)

**3. Do you currently have a dog?**

| ***** **Also include cases of people living together (including parents) and owning a dog**  ***** **Only include when a dog is living in the Study Participant’s house and/or yard**  ***** **Excluded when the dog is being raised for food or breeding purposes** |
| --- |

① Yes (→ Go to next page)

② No (→ Unable to participate in the survey)

| **1–10 The following questions gather basic personal information (General characteristics)** |
| --- |

**1. What is your gender?**

① Male

② Female

**2. What is your date of birth?**

/ (yyyy/mm)

**3. Have you ever been married?**

| ① Yes ⇨  ② No (→Go to question 4) | **3-1. If so, what is your current marital status?**  ① You and your spouse are living together  ② You have a spouse but you are not living together  ③ Your spouse has died  ④ You are divorced |
| --- | --- |

**4. Do you have a child?**

① Yes

② No

**5. How do you assess your general health?**

① Very good

② Good

③ Normal

④ Bad

⑤ Very bad

**6. What is the highest educational level you have completed?**

① Korean traditional school ② Home schooling ③ Elementary school

④ Middle school ⑤ High school ⑥ Two-year college

⑦ University ⑧ Graduate school

**7. In the past week, have you worked more than one hour for pay, or worked as an unpaid family worker for more than 18 hours?**

| ① Yes ⇨  ② No | **7-1. What type of job do you have?**  ① Employed  ② Self-employed  ③ Help family |
| --- | --- |

**8. How many people live in your household, not including yourself? _______**

**9. What is your household’s total approximate income for one year?**

Period ① Annual ② Monthly

Income (Korean Won)

**10. Which of the following types of houses do you live in?**

① Detached

② Apartment

③ Multifamily house

④ Studio apartment

| **1–18 The following questions are about your pet** |
| --- |

1. When did you get your pet?

① Before I was 6 years old

② When I was between 6 and 12 years of age

③ When I was between 13 and 18 years of age

④ After I was 19 years of age

2. Do you have any other species of pets beside your pet dog?

| ① Yes  ② No |  |
| --- | --- |

3. How many dogs do you currently have? ( )

| *** Also include cases of people living together (including parents) and owning a dog**  *** Only include when the dog is living in the house (including the yard)**  *** Excluded when being raised for food or for breeding purposes** |
| --- |

* In question 3, if you have more than one dog, please answer the questions for the oldest pet dog.

4. How old is your dog? ( )

5. How many years have you lived with your dog? ( )

6. Is your dog a pure breed or a cross breed?

| ① Pure breed ⇨ | **6–1.** What breed is your dog? ( ) |
| --- | --- |
|  |  |
| ② Cross breed ⇨ | **6-2.** If your dog is a mixed breed, what breeds is it? (If you don’t know, please answer “unknown”) |

7. What is the gender of your dog?

① Male ② Female

8. Did your dog were altered?

① Yes ②No

9. What does your dog weigh? ( kg)

10. How tall is your dog (length from the foot to the shoulder)? ( cm)

11. What is the size of your dog?

① Miniature (< 2 kg) ② Small (2–10 kg) ③ Medium (11–25 kg)

④ Large (26–50 kg) ⑤ Giant (> 50 kg)

12. How often do you walk your dog?

① More than once a day

② Once every two or three days

③ Once every four to six days

④ Less than once a week

⑤ I never walk my dog

13. How long do you spend walking with your dog usually? ( min per day)

14. On average, how many hours a day do you spend with your pet? ( hour per day)

*Including walking

15. How many times did you visit an animal hospital in the past year? ( times)

16. If your dog visited a veterinary hospital due to illness in the past year, please mark all the reasons for visiting the hospital.

| □ Dentistry | e.g., Gingivitis, Plaque, Tartar |
| --- | --- |
| □ Ophthalmology | e.g., Glaucoma, Cataracts, Corneal ulcers |
| □ Respiratory disease | e.g., Pneumonia, Tracheal collapse |
| □ Cardiology | e.g., Patent Ductus Arteriosus, Arrhythmias |
| □ Digestive disease | e.g., Diarrhea, Colitis, Small intestinal malabsorption |
| □ Orthopedic surgery | e.g., Hip dysplasia, Fractures |
| □ Cancer | e.g., Lymphoma, Mast cell tumors, Hemanglosarcoma |
| □ Dermatology | e.g., Allergies, Ear diseases |
| □ Neurology | e.g., Myositis, Masthenia Gravis |
| □ Heartworm |  |
| □ Vaccination |  |
| □ Emergency |  |
| □ Other |  |

17. The Body Condition Score (BCS) chart is presented next. What is your dog’s BCS? ( )


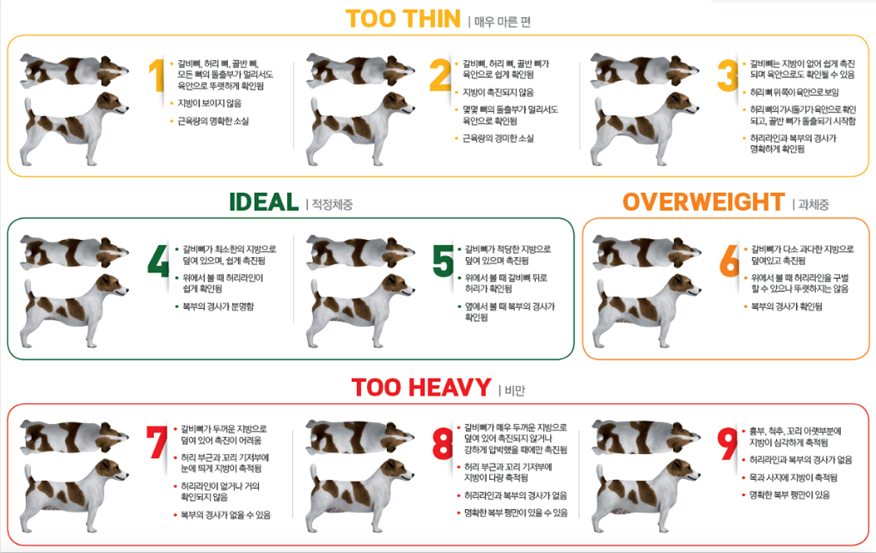


18. How did you get your dog?

① Adopted from a shelter

② Strayed in

③ Pet shop

④ Breeder

⑤ Friend or gifted

⑥ Born in the house

⑦ Veterinary Surgeon

⑧ Internet

⑨ Other

| **1–18 The following statements are used to assess Study Participants’ Pet Attachment Scores** |
| --- |

1. I really like seeing pets enjoy their food.

① strongly disagree ② moderately disagree ③ disagree

④ unsure ⑤ slightly agree ⑥ moderately agree

⑦ strongly agree

2. My pet means more to me than any of my friends (or would if I had one).

① strongly disagree ② moderately disagree ③ disagree

④ unsure ⑤ slightly agree ⑥ moderately agree

⑦ strongly agree

3. I would like to have a pet in my home.

① strongly disagree ② moderately disagree ③ disagree

④ unsure ⑤ slightly agree ⑥ moderately agree

⑦ strongly agree

4. Having pets is a waste of money.

① strongly disagree ② moderately disagree ③ disagree

④ unsure ⑤ slightly agree ⑥ moderately agree

⑦ strongly agree

5. House pets add happiness to my life (or would if I had one).

① strongly disagree ② moderately disagree ③ disagree

④ unsure ⑤ slightly agree ⑥ moderately agree

⑦ strongly agree

6. I feel that pets should always be kept outside.

① strongly disagree ② moderately disagree ③ disagree

④ unsure ⑤ slightly agree ⑥ moderately agree

⑦ strongly agree

7. I play with my pet every day (or would if I had one).

① strongly disagree ② moderately disagree ③ disagree

④ unsure ⑤ slightly agree ⑥ moderately agree

⑦ strongly agree

8. I have occasionally communicated with my pet and understood what it was trying to express (or would if I had one).

① strongly disagree ② moderately disagree ③ disagree

④ unsure ⑤ slightly agree ⑥ moderately agree

⑦ strongly agree

9. The world would be better place if people stopped spending so much time caring for their pets and started caring more for other human beings instead.

① strongly disagree ② moderately disagree ③ disagree

④ unsure ⑤ slightly agree ⑥ moderately agree

⑦ strongly agree

10. I like to feed animals out of my hand.

① strongly disagree ② moderately disagree ③ disagree

④ unsure ⑤ slightly agree ⑥ moderately agree

⑦ strongly agree

11. I love pets.

① strongly disagree ② moderately disagree ③ disagree

④ unsure ⑤ slightly agree ⑥ moderately agree

⑦ strongly agree

12. Animals belong in the wild or in zoos, but not in the home.

① strongly disagree ② moderately disagree ③ disagree

④ unsure ⑤ slightly agree ⑥ moderately agree

⑦ strongly agree

13. If you keep pets in the house you can expect a lot of damage to furniture.

① strongly disagree ② moderately disagree ③ disagree

④ unsure ⑤ slightly agree ⑥ moderately agree

⑦ strongly agree

14. I like house pets.

① strongly disagree ② moderately disagree ③ disagree

④ unsure ⑤ slightly agree ⑥ moderately agree

⑦ strongly agree

15. Pets are fun but it’s not worth the trouble of owning one.

① strongly disagree ② moderately disagree ③ disagree

④ unsure ⑤ slightly agree ⑥ moderately agree

⑦ strongly agree

16. I frequently talk to my pets (or would if I had one).

① Strongly disagree ② moderately disagree ③ disagree

④ unsure ⑤ slightly agree ⑥ moderately agree

⑦ strongly agree

17. I hate animals.

① strongly disagree ② moderately disagree ③ disagree

④ unsure ⑤ slightly agree ⑥ moderately agree

⑦ strongly agree

18. You should treat your house pets with as much respect as you would a human member of your family.

① strongly disagree ② moderately disagree ③ disagree

④ unsure ⑤ slightly agree ⑥ moderately agree

⑦ strongly agree
